# Oleic Acid and Hydroxytyrosol Inhibit Cholesterol and Fatty Acid Synthesis in C6 Glioma Cells

**DOI:** 10.1155/2017/9076052

**Published:** 2017-12-24

**Authors:** Paola Priore, Antonio Gnoni, Francesco Natali, Mariangela Testini, Gabriele V. Gnoni, Luisa Siculella, Fabrizio Damiano

**Affiliations:** ^1^Laboratory of Biochemistry and Molecular Biology, Department of Biological and Environmental Sciences and Technologies, University of Salento, Via Prov.le Lecce-Monteroni, 73100 Lecce, Italy; ^2^CNR-NANOTEC, Institute of Nanotechnology c/o Campus Ecotekne, University of Salento, Via Monteroni, 73100 Lecce, Italy; ^3^Department of Basic Medical Sciences, Neurosciences and Sense Organs, University of Bari “Aldo Moro”, Policlinico P.zza G. Cesare 11, 70100 Bari, Italy

## Abstract

Recently, the discovery of natural compounds capable of modulating nervous system function has revealed new perspectives for a healthier brain. Here, we investigated the effects of oleic acid (OA) and hydroxytyrosol (HTyr), two important extra virgin olive oil compounds, on lipid synthesis in C6 glioma cells. OA and HTyr inhibited both de novo fatty acid and cholesterol syntheses without affecting cell viability. The inhibitory effect of the individual compounds was more pronounced if OA and HTyr were administered in combination. A reduction of polar lipid biosynthesis was also detected, while triglyceride synthesis was marginally affected. To clarify the lipid-lowering mechanism of these compounds, their effects on the activity of key enzymes of fatty acid biosynthesis (acetyl-CoA carboxylase-ACC and fatty acid synthase-FAS) and cholesterologenesis (3-hydroxy-3-methylglutaryl-CoA reductase-HMGCR) were investigated in situ by using digitonin-permeabilized C6 cells. ACC and HMGCR activities were especially reduced after 4 h of 25 *μ*M OA and HTyr treatment. No change in FAS activity was observed. Inhibition of ACC and HMGCR activities is corroborated by the decrease of their mRNA abundance and protein level. Our results indicate a direct and rapid downregulatory effect of the two olive oil compounds on lipid synthesis in C6 cells.

## 1. Introduction

Extra virgin olive oil (EVOO), the principal source of fat in the Mediterranean diet, represents the topic of many studies because several epidemiological data suggest that it positively affects human health, reducing the incidence of cancer, hypertension, and cardiovascular diseases [1, 2]. Besides these well-recognized effects, recent clinical studies support the efficacy of the Mediterranean diet and of its main fat also against the cognitive decline associated with ageing as well as against the onset and progression of a number of neurodegenerative diseases. In such neurological contexts, several data highlight the role of the natural compounds whose EVOO is rich [3, 4].

The EVOO health effects can be ascribed to a plethora of molecules contained in both its saponifiable (fatty acids) and unsaponifiable (mainly cholesterol) fractions. Initially, the beneficial properties of EVOO were attributed to its high content in oleic acid (OA). OA consumption was claimed to promote cardiovascular disease prevention and to influence the expression of homeostatic and metabolic genes that protect tissues from oxidative and inflammatory processes associated with ageing, degenerative diseases, and cancer [5–9]. In a previous study carried out on rat C6 glioma cells [5], it has been shown that, among a number of various fatty acids, OA was the most effective downregulator of lipid synthesis.

In the last decade, a flurry of scientific findings has highlighted that many of the EVOO beneficial effects can be accounted not only for the monounsaturated nature of its predominant fatty acid OA but also for the bioactivity of EVOO minor compounds, which can act on cells through both indirect and direct mechanisms, the latter modulating gene expression [6, 8–10]. Among the minor constituents of EVOO, the phenolic compound hydroxytyrosol (2,(3,4-dihydroxyphenyl)-ethanol, HTyr) is considered one of the most effective antioxidants. Consumption of HTyr has certain health benefits, and the responsible mechanisms for these effects have been mainly attributed to its ability to scavenge reactive oxygen species and to enhance endogenous antioxidant systems [11]. In previous studies carried out on rat primary hepatocytes [12, 13], we found that HTyr (alone or as a part of a crude extract) stands out for its capacity to modulate also some enzyme activities relevant for lipid metabolism.

After white adipose tissue, the brain is the organ with the highest lipid content of the body. The biosynthesis and deposition of lipids play a pivotal role in maintaining the brain structure and function. Alterations in lipid metabolism are the cause of or are associated with many neurological diseases [14, 15].

Attenuation of age- and disease-associated cognitive decline by olive oil or by its minor compounds has been observed in cellular, animal, and human models [16–18]. In most of these studies, a reduction of oxidative damage and a modulation of antioxidant defences were shown. However, besides the antioxidant and anti-inflammatory activity, other mechanisms might underlie the beneficial effects of EVOO minor compounds on brain development and homeostasis [3, 16–18].

Considering the need for chemotherapeutic intervention against neurological disorders [19] and the putative role of fatty acids and antioxidants in this field [7, 15, 20], further studies are required to gain insight into the impact of EVOO compounds on neurodegenerative processes. C6 glioma cells present a large repertoire of astrocyte-expressing enzymatic activities [21, 22] and exhibit a prevalent astrocyte-like phenotype when cultured in serum-rich medium [23]. Thus, they are considered a useful cellular model to study cerebral dysfunction [20, 24].

To our knowledge, this is the first study in which the effect of EVOO constituents on lipid metabolism in glial cells has been investigated. In detail, this study was focused on the effects of the cosupplementation of OA and HTyr on cholesterol and fatty acid syntheses in glial cells.

## 2. Materials and Methods

### 2.1. Materials

Rat C6 glioma cells were from the American Type Culture Collection. Dulbecco's modified eagle's high-glucose medium (DMEM), foetal bovine serum (FBS), penicillin/streptomycin, phosphate buffer solution (PBS), and 3-[4,5-dimethylthiazol-2-yl]-2,5-diphenyltetrazolium bromide (MTT) were obtained from Gibco-Invitrogen Ltd. (Paisley, UK); [1-^14^C]acetate was obtained from GE Healthcare (Little Chalfont, UK); [1-^14^C]acetyl-CoA, [3-^14^C]3-hydroxy-3-methylglutaryl-CoA ([3-^14^C]HMG-CoA) were obtained from PerkinElmer (Boston, MA). Primary antibodies for acetyl-CoA carboxylase (ACC), fatty acid synthase (FAS), and *α*-tubulin were obtained from Cell Signaling Technologies (Boston, MA). The antibody against 3-hydroxy-3-methylglutaryl-CoA reductase (HMGCR) as well as horseradish peroxidase-conjugated IgGs was obtained from Santa Cruz Biotechnology (Dallas, TX). All other reagents, obtained from Sigma-Aldrich, were of analytical grade.

### 2.2. Cell Culture

C6 cells were maintained in DMEM supplemented with 10% FBS and 1% penicillin/streptomycin, at 37°C in a humidified atmosphere of 5% CO_2_. Unless otherwise specified in the text, C6 cells were seeded in 6-well plates (Corning Inc., Corning, NY) at a density of 5 × 10^5^ cells per well and cultured in 10% FBS-supplemented medium. 24 h after plating, the medium was changed and, following further 24 h, OA sodium salt and HTyr were added for 4 h, singularly or in coincubation, to the DMEM medium, obtaining 25 *μ*M final concentration. OA stock solution was 10 mM in DMEM and HTyr stock solution was 100 mM dissolved in dimethyl sulfoxide (DMSO). For each determination, untreated control cells were also considered.

### 2.3. Cell Viability Assay

Cell proliferation and viability were assessed by the MTT assay. To this purpose, C6 cells were cultured at a density of 5 × 10^3^ cells/well in a 96-well plate (Corning Inc., Corning, NY) and after 24 h, the serum-rich medium was refreshed. Following further 24 h, cells were incubated for 4 h with OA and/or HTyr, using for each sample three concentrations: 25 *μ*M, 50 *μ*M, and 100 *μ*M of OA and/or the HTyr. Then, cell monolayers were incubated for 3 h with 1 mg/mL MTT. Mitochondria of living cells transform the yellow-coloured tetrazolium compound to its purple formazan derivative. Formazan crystals formed in the cells were dissolved in 100 *μ*L DMSO, and the absorbance was measured at 570 nm using a Multiskan FC ELISA reader (Thermo Fisher Scientific, Waltham, MA). The viability is calculated as percentage of absorbance relative to control cells.

### 2.4. Rate of Fatty Acid and Cholesterol Synthesis

Acetyl-CoA is the precursor for both fatty acid and cholesterol synthesis. Lipogenic activity was monitored by the incorporation of [1-^14^C]acetate (16 mM, 0.96 mCi/mol) into total fatty acids and cholesterol essentially as in Gnoni et al. [25]. Cells were incubated for 4 h with 25 *μ*M OA and/or 25 *μ*M HTyr. Labelled acetate was added 1 h before ending the experiment.

To terminate the lipogenic assay, the medium was aspirated and the cells were washed three times with ice-cold PBS to remove unreacted acetate, and the reaction was stopped by 1.5 mL of 0.5 N NaOH.

The cells were scraped off, transferred to a test tube, and saponified with ethanolic KOH. Sterols and fatty acids were extracted and counted for radioactivity as reported [25].

### 2.5. Chromatographic Analysis of Radiolabelled Lipid Fractions

Radiolabelled acetate incorporation into phospholipids and neutral lipids was analyzed. At the end of the incubation period, the cells were washed with ice-cold PBS and the reaction was blocked with 2 mL of KCl:CH_3_OH (1 : 2, *v*/*v*). Total lipids were extracted according to Bligh and Dyer [26] and resolved by thin layer chromatography on silica gel plates, using as developing system CHCl_3_ : CH_3_OH : 28% NH_4_OH (65 : 25 : 4) and exane : ethyl ether : acetic acid (80 : 20 : 1) for phospholipids and neutral lipid analysis, respectively. Lipid spots were visualized with iodine vapour and scraped into counting vials for radioactivity measurement [5].

### 2.6. Chromatographic Analysis of Radiolabelled Fatty Acids

HPLC analysis of the extracted fatty acids was performed as reported [5]. 20 *μ*L of sample was injected into a Beckman Coulter System Gold Programmable Solvent Module 125 and furnished with a C18 ODS column (4.6 × 250 mm) and Diode Array Detector 168 (Beckman Coulter, Milan, IT). Two mobile phases were used for elution: solvent A, constituted by acetonitrile : water (4 : 1) and ran for 45 min and solvent B, constituted by acetonitrile and ran for another 15 min. Flow rate was 2 mL/min and detection was at 242 nm. Eluted fractions were collected for radioactivity measurement.

### 2.7. Assay of Lipogenic Enzyme Activities

ACC activity was determined as the incorporation of radiolabelled acetyl-CoA into fatty acid in an assay coupled with FAS activity. This method circumvents interferences linked to the classical bicarbonate assay [27]. ACC and FAS activities were determined in digitonin-permeabilized C6 cells. Cell permeabilization, achieved by using an assay mix containing 400 *μ*g/mL digitonin [5], represents an appropriate tool to investigate enzyme activities directly in situ (i.e., in a more or less natural environment).

FAS activity was assayed by measuring the incorporation of [1-^14^C]acetyl-CoA into fatty acids essentially as described above for ACC activity, except that 0.2 mM malonyl-CoA was included and ATP, butyryl-CoA and FAS were omitted in the digitonin-containing assay mixture [28]. The assay was carried out at 37°C for 10 min.

Both the lipogenic assays were stopped by the addition of 100 *μ*L of 10 M NaOH. The samples were saponified by adding 5 mL of CH_3_OH and boiling for 45–60 min in capped tubes. After acidification with 200 *μ*L of 12 M HCl, fatty acids were extracted and counted for the radioactivity as in [5].

The activities of ACC and FAS are expressed as nanomoles of [1-^14^C]acetyl-CoA incorporated into fatty acids per minute per milligram of protein.

### 2.8. Activity Assay of 3-Hydroxy-3-Methylglutaryl-CoA Reductase (HMGCR)

HMGCR is the rate-controlling enzyme in the biosynthesis of cholesterol. The HMGCR activity assay was carried out essentially as described in [5]. Briefly, C6 cells were seeded at a density of 2 × 10^6^ cells per 100 mm diameter Petri dish. After 48 h, 25 *μ*M OA and HTyr were added singularly or in coincubation to the medium for 4 h. Then, the medium was discarded and the cells were scraped into a buffer containing 50 mM Tris-HCl (pH 7.4) and 150 mM NaCl. After centrifugation (900 ×g, 3 min, room temperature), the pellet was frozen in liquid nitrogen and kept at −80°C until use.

Cell extracts were prepared from the pellets which were thawed, resuspended, and subsequently used for HMGCR activity assay [5]. The reaction started by the addition of [3-^14^C]HMG-CoA (75 *μ*M, 1.8 Ci/mol). After 120 min at 37°C, the reaction was stopped by the addition of 20 *μ*L of 7 M HCl. Conversion to mevalonolactone occurred following an additional 60 min incubation at 37°C and the radioactive product was isolated by TLC, using toluene : acetone (1 : 1) as the mobile phase. Spots were collected and counted for the radioactivity. As internal standard, [^3^H]mevalonolactone was used.

### 2.9. Isolation of RNA from C6 Cells and Real-Time qPCR Analysis

Total RNA from C6 cells was isolated using the SV Total RNA Isolation System kit (Promega), following the manufacturer's instructions. The reverse transcriptase reaction (20 *μ*l) was carried out using 5 *μ*g of total RNA, 100 ng of random hexamers, and 200 units of SuperScript III RNase H-Reverse Transcriptase (Life Technologies—Thermo Fisher Scientific, Waltham, MA) [29].

Quantitative gene expression analysis was performed using SYBR® Select Master Mix for CFX (Life Technologies—Thermo Fisher Scientific, Waltham, MA) and 18S rRNA for normalization. The primers used for quantitative real-time PCR analysis were as follows (5′ to 3′): rFASNfor CTCTGGTGGTGTCTACATTTC; rFASNrev GAGCTCTTTCTGCAGGATAG; rACCfor CTTGGAGCAGAGAACCTTCG; rACCrev; CCTGGATGGTTCTTTGTCCC; rHMGCRfor CTCACAGGATGAAGTAAGGG; rHMGCRrev CTGAGCTGCCAAATTGGACG [30]

### 2.10. Western Blotting Analysis

Cells grown in 6-well dishes were treated with OA and/or HTyr as indicated above and lysed as previously described [12]. The extracts were boiled for 5 min and samples containing an equal amount of total protein (25 *μ*g) were loaded on 10% SDS-polyacrylamide gels. Following electrophoresis, the proteins were transferred onto a nitrocellulose membrane [13]. To detect ACC, FAS, and HMGCR, membranes were incubated with the specific primary antibodies for 1.5 h at room temperature and then for 1 h with appropriate horseradish peroxidase-conjugated IgG (dilution 1 : 5000). Signals were detected by enhanced chemiluminescence using the Amersham ECL plus kit (GE Healthcare, Milan, Italy). Beta-actin detection was used for signal normalization.

## 3. Statistical Analysis

Data are the means ± S.D. for the indicated number of experiments. The results were computed with Excel (Microsoft 10). Comparisons among groups were made using one-way analysis of variance (ANOVA). The differences between mean values were tested, using Bonferroni post hoc test. All statistical analyses were performed by GraphPad Prism 6 software (GraphPad Software Inc., La Jolla, CA). Differences were considered statistically significant at *P* < 0.05.

## 4. Results

### 4.1. Cell Viability

**Figure 1:**
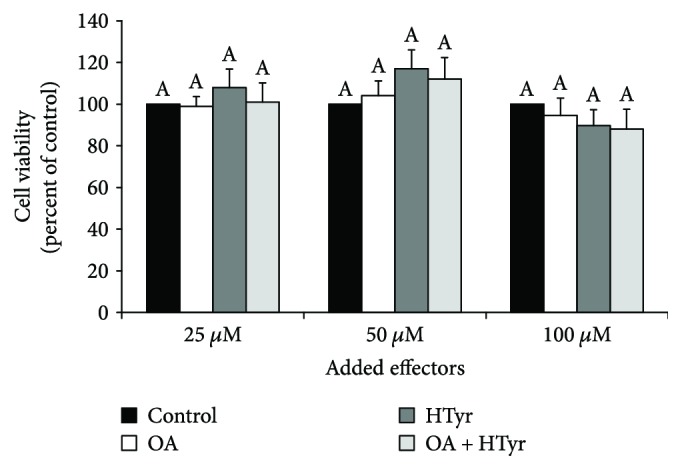
Effect of oleic acid and/or hydroxytyrosol on C6 cell viability. C6 cells were incubated for 4 h with 25 *μ*M, 50 *μ*M, and 100 *μ*M OA, HTyr, or their combination in serum-rich medium. Cell viability was estimated by an MTT assay. Values, expressed as % of control, are means ± S.D. of five experiments. Within the same group, samples bearing different letters differ significantly (*P* < 0.05). Control: untreated cells; OA: oleic acid; HTyr: hydroxytyrosol.

MTT test showed that C6 cells incubated with OA or HTyr (25 *μ*M, 50 *μ*M, and 100 *μ*M for 4 h), had the same viability of the control cells at each tested concentration (Figure 1). Also, the coincubation of OA and HTyr did not exert any cytotoxic effect. These findings were corroborated by morphological observation, protein assay, and trypan blue exclusion (data not shown). Thus, all further experiments were performed on cells treated with 25 *μ*M OA, 25 *μ*M HTyr, or their combination and incubated for 4 h in order to exclude putative nonspecific toxic effect while at the same time using the lowest effective concentration.

### 4.2. Effect of OA, HTyr, and Their Combination on Cholesterol and Fatty Acid Syntheses

Acetate in the cell is transformed into acetyl-CoA, which represents a common precursor for both fatty acid and cholesterol synthesis. Hence, both these metabolic pathways were simultaneously followed by using labelled acetate as a precursor.

**Figure 2:**
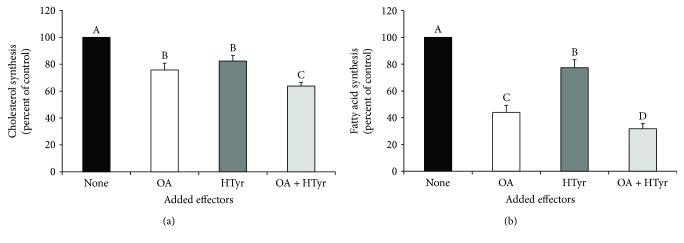
Modulation of cholesterol and fatty acid syntheses by oleic acid and/or hydroxytyrosol. After an initial 48 h plating, C6 glioma cells, growing in serum-rich medium, were incubated for 4 h with 25 *μ*M OA and/or 25 *μ*M HTyr. During the last hour of incubation, labelled acetate was added and its incorporation into cholesterol (a) and fatty acids (b) was followed. Data, nmol [1-^14^C]acetate/h/mg protein, are expressed as % of control and are means ± S.D. of six independent experiments. In each experiment, determinations were carried out in triplicate. In control cells, rates of cholesterol and fatty acid synthesis were 1.43 ± 0.07 and 8.67 ± 0.49 nmol [1-^14^C]acetate inc/h/mg protein, respectively. Samples bearing different letters differ significantly (*P* < 0.05). None: no addition to the cells; OA: oleic acid; HTyr: hydroxytyrosol.

Bar graphs in Figure 2 show a significant reduction of [1-^14^C]acetate incorporation into total cholesterol (Figure 2(a)) and fatty acids (Figure 2(b)). In particular, when C6 cells were incubated for 4 h with OA or HTyr, a decrease by 24% and 18%, respectively, of [1-^14^C]acetate incorporation into cholesterol was observed. This inhibition was much more evident (−36% versus untreated cells) if OA and HTyr were added in combination to the cells.

With respect to cholesterologenesis, fatty acid synthesis was greater affected by EVOO compounds under investigation. Incubation of C6 cells singularly with OA, or HTyr led to a reduction of the radiolabelled acetate incorporation into fatty acids by about 56% and 23%, respectively, compared to that measured in control cells. Analogously to cholesterol synthesis, fatty acid synthesis inhibition was more pronounced (−68% versus untreated cells) after 4 h of OA and HTyr coincubation of C6 cells.

### 4.3. Effect of EVOO Components on Radiolabelled Acetate Incorporation into Phospholipids and Neutral Lipids

Since newly synthesized fatty acids are mainly incorporated into complex lipids, the effect of OA and HTyr addition to C6 glioma cells on [1-^14^C]acetate incorporation into polar and neutral lipids was tested (Table 1). A general decrease of labelled precursor incorporation into all phospholipids, particularly into phosphatidylcholine, the most abundant phospholipid in C6 glioma cells, was observed mainly when cells were incubated for 4 h with OA and HTyr in combination. Among neutral lipids, unesterified fatty acids, cholesterol, and cholesterol ester were the fractions showing significant reduction in radioactivity incorporation due to the EVOO compound addition. Interestingly, only slight reduction in the incorporation of labelled acetate into triglycerides (TG) was detected after additions of OA and HTyr.

### 4.4. Analysis of Newly Synthesized Radiolabelled Fatty Acids

In order to investigate the effect of OA and HTyr additions to C6 cells on the individual fatty acids synthesized from labelled acetate, an HPLC analysis of the total fatty acid extract was carried out.

**Figure 3:**
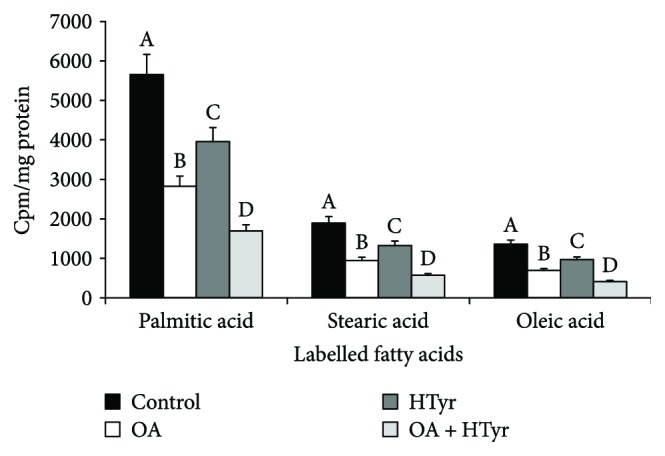
Effect of oleic acid, hydroxytyrosol, or their combination on [1-^14^C]acetate incorporation into individual fatty acids. The effects of 25 *μ*M OA, 25 *μ*M HTyr, and their combination on the incorporation of labelled acetate into different fatty acids were assayed. After 4 h of incubation, the radiolabelled neosynthesized fatty acids were extracted and separated by HPLC. Eluted fractions, corresponding to the different fatty acids, were collected for radioactivity measurement. Data, expressed as cpm/mg protein, represent means ± S.D. of six experiments. Within the same group, samples bearing different letters differ significantly (*P* < 0.05). OA: oleic acid; HTyr: hydroxytyrosol.

Figure 3 shows that, in agreement with previous results [5], in control cells, the incorporation of labelled acetate into the individual fatty acids was in the following order: palmitic acid (C16:0) > stearic acid (C18:0) > oleic acid (C18:1). Only a small amount of radioactivity was incorporated into other fatty acids (data not shown). A reduction of about 50% of the radiolabelled incorporation into palmitic, stearic, and oleic acid was observed upon OA addition to the cells, while a near 30% decrease was evidenced upon HTyr treatment. The inhibitory effect of [1-^14^C]acetate incorporation into the fatty acids was more pronounced (about 70%) when both OH and HTyr were contemporaneously added to the medium culture.

### 4.5. Modulation of Activity of ACC, FAS, and HMGCR by OA, HTyr, and Their Combination

Palmitic acid and to a lesser extent stearic acid are the main final products of the de novo fatty acid synthesis.

In order to determine the enzymatic steps of lipid biosynthetic pathways affected by the addition of OA, HTyr, and their combination, experiments were carried out to assay the activities of the key enzymes ACC and FAS for de novo fatty acid synthesis and HMGCR for cholesterol synthesis. All enzymatic activities were measured by in situ assays using digitonin-permeabilized C6 cells.

**Figure 4:**
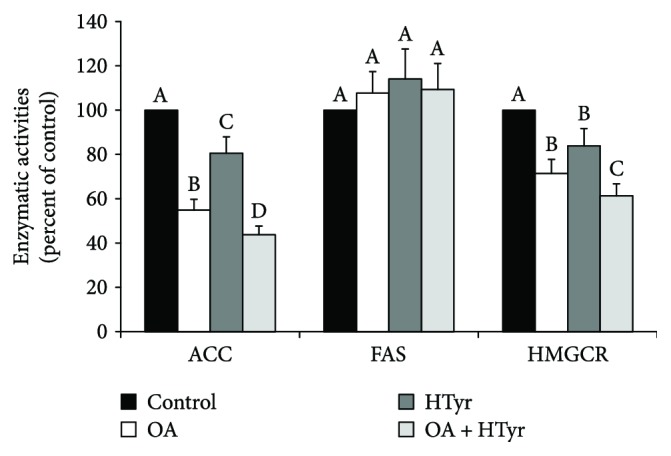
Oleic acid and/or hydroxytyrosol modulation of ACC, FAS, and HMGCR activities. After 4 h incubation with 25 *μ*M of OA, HTyr, or OA + HTyr, the indicated enzyme activities were assayed in digitonin-permeabilized C6 cells. Values, expressed as percentage of control, are means ± SD of five independent experiments. Control-specific activities were ACC, 0.178 ± 0.011 [1-^14^C]acetyl-CoA inc/min/mg protein; FAS, 0.051 ± 0.003 nmol [1-^14^C]acetyl-CoA inc/min/mg protein; HMGCR, 33.6 ± 1.9 pmol [3-^14^C]HMG-CoA inc/min/mg protein. Within the same group, samples bearing different letters differ significantly (*P* < 0.05). OA: oleic acid; HTyr: hydroxytyrosol.

4 h incubation of C6 cells with 25 *μ*M of OA or HTyr caused a reduction of ACC activity by about 45% and 19%, respectively (Figure 4). A further decrement (−56% versus untreated cells) was measured when these compounds were coincubated.

OA or HTyr lowered HMGCR activity by 29% and 16%, respectively. During coincubation, the inhibition was more pronounced. In fact, HMGCR activity reached 61% of that observed in control cells in presence of 25 *μ*M OA and 25 *μ*M HTyr.

Notably, FAS activity was not significantly affected by either incubation conditions.

The reduced activity of ACC and of HMGCR is in accordance with the results of Figure 2, regarding the reduction of total synthesis of fatty acids (Figure 2(a)) and cholesterol (Figure 2(b)) starting from [1-^14^C]acetate.

### 4.6. Regulation of ACC, FAS, and HMGCR Expression by OA, HTyr and OA + HTyr

Next, the molecular mechanisms responsible for the modulation exerted by OA or HTyr on the above-reported enzyme activities were investigated. To this aim, the abundance of mRNAs encoding for ACC, FAS, and HMGCR was quantified by real-time qPCR analysis, and the amount of the corresponding encoded protein was determined by Western blotting.

**Figure 5:**
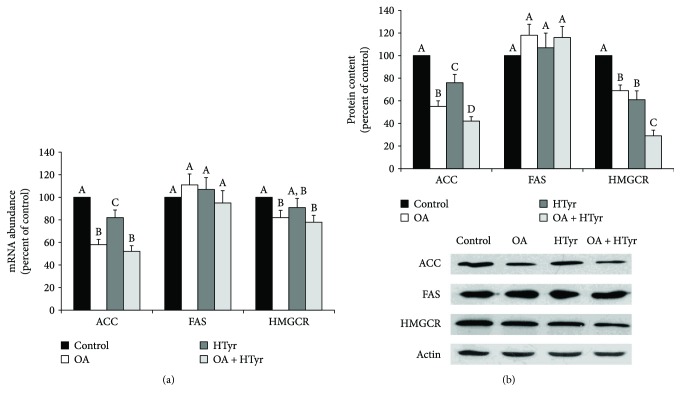
Effect of oleic acid, hydroxytyrosol, or their combination on ACC, FAS, and HMGCR mRNA abundance and protein content in C6 glioma cells. C6 cells were incubated with 25 *μ*M of OA, HTyr, or OA + HTyr for 4 h in serum-rich medium. (a) The abundance of ACC, FAS, and HMGCR mRNA was determined by RT-qPCR and normalized with respect to 18S rRNA, used as a reference. Normalized values are expressed in histograms as percentage of the control. Values are means ± S.D. of triplicate samples from each of four independent experiments. (b) C6 cells were lysed and protein content was isolated. ACC, FAS, and HMGCR were then assessed by Western blotting and quantified by densitometry. The protein contents are expressed as percentage of the control and are means ± SD of three independent experiments. Within the same group, samples bearing different letters differ significantly (*P* < 0.05). OA: oleic acid; HTyr: hydroxytyrosol.

In treated cells, a decrease of ACC mRNA abundance was observed (Figure 5(a)). After OA incubation, ACC mRNA level decreased by about 42% with respect to that measured in control cells; HTyr singularly added to the cell culture medium exerted minor inhibitory effect. In all the experimental conditions tested, no significant change in the abundance of FAS mRNA was detected (Figure 5(a)). After 4 h incubation with 25 *μ*M OA alone or in combination with 25 *μ*M HTyr, the amount of HMGCR mRNA lowered by about 18% and 22%, respectively (Figure 5(a)). Results obtained for ACC, FAS, and HMGCR mRNA abundance were confirmed also by the Western blotting analysis of the respective protein contents (Figure 5(b)).

## 5. Discussion

The Mediterranean diet has been considered the healthier dietary regimen, related to a reduced risk of several pathologies such as metabolic disorders and cardiovascular diseases [1, 2, 6, 31, 32]. A key component of the Mediterranean diet is the EVOO, which is characterized by the presence of bioactive phytonutrients, with antioxidant and anti-inflammatory properties, and of monounsaturated fatty acid (OA) as fat source.

Studies from a number of research groups have established that variations in dietary fatty acids or in the intake of phenolic compounds are able to influence cellular metabolism and regulatory processes in neuronal and glial cells [3, 5, 17, 24], supporting mounting evidence that EVOO active components could have a great potential to reduce the incidence of neurodegenerative diseases [3, 18, 33]. It has to be stressed that most of these studies focused mainly on the antioxidant and anti-inflammatory activities of fatty acids [7] and of phenolic compounds [20], without taking into account their possible direct action on metabolism, such as lipid biosynthesis.

Lipids are fundamental components of neuronal membranes and are essential for brain function. Cholesterol and fatty acids are particularly present in the synaptic membranes and play a key role in the membrane fluidity and in the formation of specialized microdomains, lipid rafts, essential for synaptic transmission [34].

Brain lipids can derive from blood and/or can be endogenously synthesized. Fatty acids can cross the blood-brain barrier by a complex process, involving diffusional and protein-mediated transport, which occurs predominantly by fatty acid transport protein-1 and protein-4 (FATP-1 and FATP-4) in humans and mouse [34]. Lipid synthesis is inefficient in neurons but not in glia cells. *In vitro* studies have demonstrated that astrocytes, the most abundant glia cells, synthesize and release lipids that are complexed to apolipoprotein E- (ApoE-) containing lipoproteins [35]. Consequently, neuronal functions are negatively affected by perturbed lipid metabolism which has been observed in several neurological disorders, such as Niemann-Pick's disease [36], Alzheimer's disease [37], Huntington's disease [38], Parkinson's disease [39], and amyotrophic lateral sclerosis [40].

Despite the crucial role of EVOO bioactive compounds in brain function and metabolism, little is known about their action on lipogenesis in glial cells [5].

This work primarily shows that both fatty acid and cholesterol syntheses are rather active in cultured glioma C6 cells when [1-^14^C]acetate is used as common precursor for both the metabolic pathways, thus adding further support to previous findings [5]. Notably, in human malignant glial cells, compared with their normal counterparts, a very active de novo fatty acids and cholesterol syntheses have been reported [41].

The present study represents the first report of a direct and rapid effect of EVOO main compounds (OA and HTyr) on lipid synthesis in rat glioma cells. We show that in C6 cells, the addition of OA and HTyr causes a fast (within 4 h) inhibition of radiolabelled acetate incorporation into both cholesterol and fatty acid fractions. A rapid effect of EVOO components on both these pathways has been described in different cell cultures [12, 13]. Beside the OA, a monounsaturated fatty acid, which represents the most abundant fatty acid (about 70%) in EVOO, polyunsaturated fatty acids (such as linoleic acid), and saturated fatty acid (mainly palmitic acid) are significantly present in the fatty acid fraction of EVOO. However, Natali et al. reported that OA was the most effective in reducing lipid synthesis in C6 cells [5].

Compared to control cells, the radiolabelled precursor incorporation into cholesterol decreased by about 30% in cells incubated with 25 *μ*M OA and 25 *μ*M HTyr (Figure 2(a)). This result can be explained, at least in part, by the reduction of HMGCR activity (Figure 4) and the regulatory enzyme of cholesterol synthesis, which is reasonably related to changes in the HMGCR expression. Indeed, the expression of HMGCR at both mRNA and protein levels was noticeably reduced in C6 cells upon treatment with both OA and HTyr.

However, the reduction of cholesterologenesis by EVOO active compounds was often less pronounced, compared with fatty acid synthesis, especially in the case of the OA and HTyr coincubation.

Incubation of C6 cells with OA or HTyr singularly or in combination caused a reduction of [1-^14^C]acetate incorporation into fatty acids and of their subsequent esterification into complex lipids. The strongest inhibition of labelled acetate incorporation into the phospholipid fractions, mainly into phosphatidylcholine, was observed upon OA and HTyr coincubation (Table 1).

Unlike phospholipids, TG synthesis in C6 cells seems to be poorly affected by EVOO compounds. The opposite trend has been reported to occur in rat hepatocytes, where EVOO components, almost without effect on phospholipid synthesis, greatly reduced acetate incorporation into TG [12, 13]. These findings are in agreement with the role of hepatic cells in TG synthesis [42] and with the assumption that dietary phenolic compounds may have a protective role against hepatic steatosis [12, 13]. Phospholipids and cholesterol are important components of biological membranes. Thus, the remarkable decrease we observed in the present study of the phospholipids and cholesterol syntheses exerted by OA and HTyr led us to suppose that EVOO components might modulate in C6 cells the shift towards membrane biogenesis instead of an accumulation of cellular neutral lipids. Actually, this hypothesis is corroborated by previous studies which indicated that fatty acids exogenously added to C6 cells may represent specific means of controlling gliomatous growth [5, 43, 44].

Coincubation with OA and HTyr greatly reduced the incorporation of [1-^14^C]acetate into the individual fatty acids, in particular into palmitic acid (Figure 3), the principal final product of the de novo fatty acid synthesis. This metabolic pathway is catalyzed by two enzymatic systems working in sequence: ACC and FAS. The activity of ACC, first committing step in fatty acid biosynthesis, was reduced in C6 cells treated with OA together with HTyr. Then, the reduced ACC activity in OA-/HTyr-treated C6 cells could explain, at least in part, the decrease in the [1-^14^C]acetate incorporation into the whole fatty acid fraction observed in Figure 2. The molecular mechanism of this reduction was deepened, and our results clearly indicate that coincubation of OA and HTyr reduced either ACC mRNA abundance or protein level. Conversely, in our experimental condition, that is, 4 h of treatment, OA and/or HTyr incubation showed practically no effect on FAS activity and on FAS mRNA abundance and protein level. With regard to the apparent insensitivity of FAS to 4 h OA and HTyr incubation, it is worth noting that while ACC is regulated by both short- and long-term mechanisms, only the latter is involved in FAS modulation [12, 45].

A great body of evidence indicates HTyr as a potent antioxidant, and its ability to decrease reactive oxygen species (ROS) in both *in vitro* and *in vivo* experiments has been well documented (for review, see [46]). Recent works have highlighted that ROS promote the expression of sterol regulatory element-binding proteins (SREBPs) [47, 48] and transcription factors involved in the upregulation of key enzymes of lipogenesis and cholesterologenesis [31, 49, 50]. In fly, treatment with an antioxidant reduces ROS, suppresses lipid droplet accumulation, and delays neurodegeneration [48]. The present study shows that, in addition to the reported antioxidant effect, HTyr supplementation to C6 cells determines an early and direct decreasing effect on fatty acid synthesis and cholesterologenesis. This effect can be at least partially ascribed to a molecular mechanism that involves the downregulation of the expression of ACC and HMGCR. This decrease is more evident when OA is added to the cells together with HTyr, indicating in most cases an additive effect of these EVOO components on lipid metabolism. Thus, also this aspect should be considered in determining the beneficial action of EVOO components, OA and HTyr, in brain dysfunction when alterations of lipid metabolism take place.

An important matter concerns the bioavailability of EVOO phenolic compounds. HTyr is dose-dependently absorbed in humans [51, 52]. Data on plasma phenol concentrations that can be achieved in humans after consumption of olive oil are poor and controversial [52]. This may be due to a number of factors: (i) the very variable levels of phenols found in EVOO (50–800 mg/kg) [53], (ii) the level of dietary intake of olive oil, and (iii) the method and the time chosen for plasma phenol quantification [53]. Most of HTyr is present in plasma and urine in conjugated forms, mainly glucuroconjugates, suggesting extensive first-pass intestinal/hepatic metabolism of the ingested HTyr [54]. However, a plasma concentration of HTyr up to 15 *μ*M has been measured in humans during the first 4 h after ingestion of 40 mL of olive oil containing a considerable amount of phenols (366 mg/kg) [55]. Moreover, it has been established that HTyr is able to cross the blood-brain barrier, although it presents a low brain uptake [3]. Considering the above, the question of EVOO phenol bioavailability in the brain remains a matter of debate and the physiological relevance of these *in vitro* findings needs to be tested in appropriate animal models and in humans. However, even if the HTyr concentration used in the present study (25 *μ*M) could be considered a pharmacological dose, the present study indicates an early and direct downregulatory effect of HTyr on fatty acids and cholesterol syntheses.

## 6. Conclusions

Correct lipid homeostasis is essential for cell survival and performance. Thus, brain fatty acid and cholesterol synthesis are critically challenged in several neurodegenerative diseases. The modulation of the activity and expression of the key enzymes of these metabolic pathways by OA and HTyr suggest a putative role in the prevention of neurological diseases, where dysfunction of lipid metabolism is involved. In this context, the reduced ACC and HMGCR expression and activity we observed in glial cells treated with such compounds may be considered of importance.

## Figures and Tables

**Table 1:** Effect of OA and HTyr and their combination on [1-^14^C]acetate incorporation into various lipid fractions in C6 cells

Added effectors	None	OA	HTyr	OA + HTyr
*Polar lipids*				
CL + PE	2151 ± 151^a^	1377 ± 85^b^	1742 ± 121^c^	1119 ± 75^d^
PC	15,908 ± 875^a^	5596 ± 308^b^	12,781 ± 703^c^	3487 ± 192^d^
SM	741 ± 67^a^	463 ± 37^b^	640 ± 37^a^	270 ± 19^c^
PS + PI	3781 ± 246^a^	1439 ± 101^b^	2533 ± 165^c^	962 ± 65^d^
*Neutral lipids*				
MG	289 ± 20^a^	231 ± 11^b,c^	252 ± 11^b^	214 ± 7^c^
DG	711 ± 136^a^	587 ± 24^a^	623 ± 27^a^	562 ± 35^a^
Cholesterol	2155 ± 194^a^	1724 ± 155^b^	1896 ± 170^a,b^	1509 ± 136^b^
Unesterified fatty acids	239 ± 14^a^	200 ± 12^a,b^	187 ± 11^b^	123 ± 8^b^
TG	1123 ± 73^a^	1048 ± 68^a,b^	999 ± 65^a,b^	921 ± 60^b^
CE	896 ± 63^a^	659 ± 46^b,c^	725 ± 47^a,b^	587 ± 23^c^

C6 cells were incubated with 25 *μ*M oleic acid (OA) and/or 25 *μ*M hydroxytyrosol (HTyr) for 4 h, and labeled acetate was added 1 h before ending the incubation. Total lipids were extracted. Phospholipids and neutral lipids were resolved by TLC, and the radioactivity associated with the different lipid fractions was counted. CL: cardiolipin; PE: phosphatidylethanolamine; PC: phosphatidylcholine; SM: sphingomyelin; PS: phosphatidylserine; PI: phosphatidylinositol; MG: monoglycerides; DG: diglycerides; TG: triglycerides; CE: cholesterol esters. Values are expressed as cpm/mg protein ± SD, *n* = 5. Within the same group, samples bearing different letters differ significantly (*P* < 0.05).
